# Tau Protein Interaction Partners and Their Roles in Alzheimer’s Disease and Other Tauopathies

**DOI:** 10.3390/ijms22179207

**Published:** 2021-08-26

**Authors:** Jakub Sinsky, Karoline Pichlerova, Jozef Hanes

**Affiliations:** Institute of Neuroimmunology, Slovak Academy of Sciences, Dubravska Cesta 9, 845 10 Bratislava, Slovakia; jakub.sinsky@savba.sk (J.S.); karoline.pichlerova@savba.sk (K.P.)

**Keywords:** tau protein, interaction partners, Alzheimer’s disease, tauopathies

## Abstract

Tau protein plays a critical role in the assembly, stabilization, and modulation of microtubules, which are important for the normal function of neurons and the brain. In diseased conditions, several pathological modifications of tau protein manifest. These changes lead to tau protein aggregation and the formation of paired helical filaments (PHF) and neurofibrillary tangles (NFT), which are common hallmarks of Alzheimer’s disease and other tauopathies. The accumulation of PHFs and NFTs results in impairment of physiological functions, apoptosis, and neuronal loss, which is reflected as cognitive impairment, and in the late stages of the disease, leads to death. The causes of this pathological transformation of tau protein haven’t been fully understood yet. In both physiological and pathological conditions, tau interacts with several proteins which maintain their proper function or can participate in their pathological modifications. Interaction partners of tau protein and associated molecular pathways can either initiate and drive the tau pathology or can act neuroprotective, by reducing pathological tau proteins or inflammation. In this review, we focus on the tau as a multifunctional protein and its known interacting partners active in regulations of different processes and the roles of these proteins in Alzheimer’s disease and tauopathies.

## 1. Introduction

Proteins are essential macromolecules that play important roles in almost any cellular process. They usually do not function alone but rather as complexes with other molecules, mainly with proteins. Protein-protein interactions (PPIs) are elementary for many processes and it is proposed that their dysfunction or deregulation is located upstream, leading to various pathological conditions [1]. In Alzheimer’s disease and other tauopathies, tau protein undergoes pathological modifications that lead to the formation of paired helical filaments (PHF) and neurofibrillary tangles (NT) which belong to the main hallmarks of these diseases. The conversion of physiological tau into its pathological forms and their participation in disease etiology have not been fully understood yet and are still under investigation. In physiological conditions, tau interacts with many protein partners which maintain their proper structure and function. Under pathological conditions, proteins interacting with tau can also participate in its non-physiological modifications leading to the development of neurodegenerative diseases. Interaction partners of tau protein and involved molecular pathways can either initiate and drive the tau pathology or can have neuroprotective roles, by reducing pathological tau changes or inflammation.

**Figure 1:**
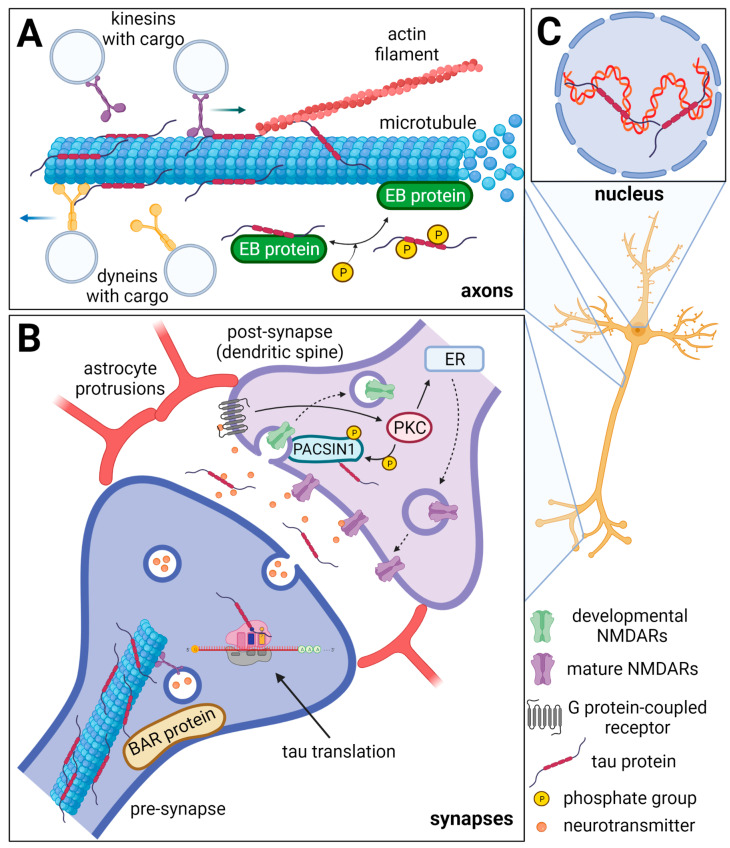
Schematic representation of physiological tau protein functions in neurons. The axonal tau (**A**) stabilizes microtubules (MTs), and it can also bind actin filaments thus facilitating cytoskeleton networking. Furthermore, tau regulates MT dynamics by interacting with end-binding (EB) proteins. The EB proteins promote and regulate MT nucleation and elongation. Tau inhibits the EB protein binding to MTs and this inhibition is reversed by tau phosphorylation. Tau also competitively inhibits the interaction of dynein and kinesin to MTs and thus influences the intraneuronal transport and cargo release. In the synapses (**B**), tau protein can be directly translated, and during neuronal activity, it is released into the synaptic cleft. Through linking the MTs and actin filaments, tau can influence synaptic plasticity. Moreover, tau is a known interacting partner of the BAR domain-containing proteins such as BAIAP2, PACSIN1, and BIN1, that ensure the curvature and shaping of the neuronal membrane. Tau may play a role in the removal of developmental NMDARs and their replacement for mature NMDARs in dendrites (dashed arrows) as it is a known substrate of the protein kinase C (PKC), which is activated by the G protein-coupled receptors (GPCR). The PACSIN1 recruits clathrin and dynamin endocytic machinery to the developmental NMDARs and thus mediates their removal. The developmental/mature NMDARs exchange is important for the formation of new synaptic connections. ER—endoplasmic reticulum. In the nucleus (**C**), tau interacts with DNA and RNA, maintains their integrity, and protects them from oxidative damage. Furthermore, tau may be involved in rRNA-coding DNA transcription and rRNA processing. Created with BioRender.com.

Tau protein belongs to the family of microtubule-associated proteins (MAPs) [2,3] and can influence axonal transport and growth [4], neuronal polarization [5], and thus the normal function of neurons and the brain (Figure 1) [6,7].

**Figure 2:**
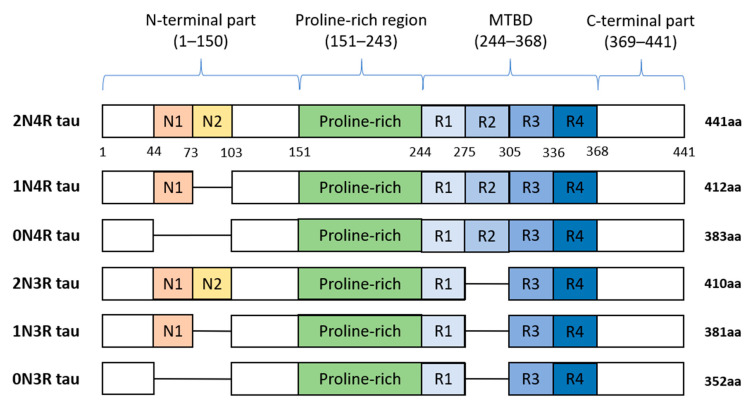
Schematic representation of six human tau protein isoforms and their domains. The N-terminal part is more acidic due to N1 and N2 inserts. The microtubule-binding domain (MTBD) is composed of repeats R1–R4. The number of repeats and N-terminal inserts varies according to the type of tau protein isoform.

In humans, tau protein is expressed mainly in neurons [8], and in lower amounts in oligodendrocytes and astrocytes [9,10,11,12]. Besides the central nervous system (CNS), tau is expressed by peripheral neurons [13], and more recently, tau immunoreactivity was also found in the human submandibular gland and sigmoid colon tissues [14]. Human tau protein is encoded by *MAPT* gene localized on chromosome 17q21 and consists of 16 exons. In the adult human brain, six tau isoforms are expressed ranging from 352 to 441 amino acids (see Figure 2). Three decades ago, it was proposed that individual tau isoforms may have different functions since they are differently expressed in the fetal and developed brain [15,16,17]. Now, it is known that alternative splicing even varies across neuronal cell types and during neuronal maturation [18,19].

Tau protein is distinctly divided into the N-terminal part, proline-rich region (PRR), microtubule-binding domain (MTBD), and C-terminus. The N-terminal domain length is dependent on alternative splicing of exons 2 and 3 which encode acidic amino acids. In MTBD, the splicing-dependent manner of exon 10 results in either 3 (3R) or 4 (4R) microtubule-binding repeats which are essential for binding of tau to individual tubulin heterodimers, and through this interaction tau stabilizes microtubules (MT) [20].

Despite the MT-stabilizing function of tau, its removal or modification had no significant impact on microtubule stability, cellular function, or cognition in mouse models [21,22,23,24,25]. It was shown that the MT-stabilizing function of tau protein is replaceable by microtubule-associated proteins MAP1a [22,26] or MAP1b [5]. However, experiments performed by several other groups on tau-knockout mice revealed that tau can influence the regulation of neuronal activity [27], synaptic plasticity [28], neurogenesis [29], iron export from neurons [30], and long-term depression of synapses [31,32]. Along with other animal disease models where the tau levels were reduced, it was found that depletion of tau protein had a protective effect on neurons against amyloid-beta (Aβ) induced excitotoxicity or by other excitotoxins in mice over-expressing amyloid precursor protein (APP) and presenilin 1 [33,34,35,36,37]. In chemically induced seizure models, hyperexcitability of neurons was reduced in mice with attenuated tau expression [38]. This suggests that endogenous tau is integral for regulating, or rather, upregulating neuronal hyperexcitability in diseased animals. In addition, in mice with impaired function of voltage-dependent sodium and potassium channels, the depletion of tau protein had a protective effect on neurons [39]. These studies suggest that tau protein may play a role in the regulation of neuronal network activity in both pathological and physiological conditions. Moreover, this is also supported by the evidence that tau protein has several additional roles in neurons and the brain [6].

In pathological conditions, the accumulation of insoluble tau aggregates occurs inside neurons, in extracellular space [40,41], and other brain cells such as astrocytes and oligodendrocytes [42,43]. The formation of this stable material is the consequence of abnormally modified and truncated tau proteins which self-aggregate and gradually mature to paired helical filaments (PHF) and neurofibrillary tangles (NFT) which are common hallmarks of several neurodegenerative diseases [44]. The formation of PHFs and NFTs is associated with the engulfment of the cytosol of neurons, failure in intracellular trafficking, and gradual disruption of basic physiological processes that end in apoptosis and neuronal death [45,46,47]. The formation of these aggregates is accompanied by inflammation which on one hand could help in their clearance, but on the other, can exacerbate the pathological processes [48,49,50]. Tau pathology is the main cause of dementia in Alzheimer’s disease and other neurodegenerative diseases, including frontotemporal dementia [51], argyrophilic grain disease [52], corticobasal degeneration [53,54], progressive supranuclear palsy [55], and several other diseases [44]. These disorders, where the accumulation of abnormal tau protein in the brain occurs, are referred to as tauopathies.

## 2. Roles of Tau Protein in Physiology and Pathology

### 2.1. Tau and Axonal Transport

Tau protein is enriched in axons where it binds microtubules through its MTBD and participates in their stabilization and regulation. Tau protein’s “free” flanking N-terminal and C-terminal regions interact with various classes of proteins involved in the regulation of cytoskeleton [56,57] and motor proteins kinesins and dyneins [58,59]. Thus, tau participates in the regulation of intraneuronal transport and modulation of microtubule dynamics, which ensures flexible reorganization of cytoskeleton and synaptic transmission. Tau can modulate functions of motor proteins by competitive inhibition of interactions of dynein and kinesin with microtubules, facilitating dynein binding to microtubules, or regulation of transport-vesicle releasing from motor proteins [60,61].

### 2.2. Tau Protein in Synapses

Besides axons, tau protein occurs also in pre- and post-synapses and in smaller amounts also in dendrites [31,32,33]. Tau protein can be directly translated in synapses and this translation is regulated by synaptic activity [62]. Furthermore, synaptic activity mediates tau protein release into extracellular space including synaptic clefts [63,64,65,66]. When discussing tau in the neuronal synapse, it is important to mention that synapses are complex biological structures also comprising astrocytes. These physiological structures composed of neuronal synapses and astrocyte protrusions are referred to as tripartite synapses [67,68]. Through these protrusions, astrocytes maintain the proper function of synapses through astrocyte–lactate shuttle, glutamate and GABA uptake from the synaptic cleft, growth factors release, and other processes [69]. In tauopathies, tau protein aggregates damage these tripartite synapses and disturb the normal function of neuronal networks [70]. Furthermore, the microglia, which constantly scan the surroundings with their processes, interact regularly with synapses [71,72]. Along with the fact that tau proteins are released during synaptic activity under both, physiological and pathological conditions, the microglia themselves also can mediate the inter-cellular spreading of the tau protein [73]. Released extracellular tau could be capable of interactions with proteins present in the synaptic clefts, thus influencing synaptic functions.

The structure of synapses is determined by the actin filaments, microtubules, and proteins modulating the membrane shape. It was shown that tau can mediate changes in the dendritic cytoskeleton and regulate synaptic plasticity and signaling [74,75]. Tau protein is known to interact with proteins regulating cytoskeleton and membrane curvature which are essential for synapse formation and sustainability [56,76,77,78,79]. Tau binds actin via its PRR, and at the same time also binds microtubules through its MTBD and serves as a crosslinker between microtubules and actin filaments and thus helps to organize the cytoskeleton network [20,80,81]. Furthermore, tau regulates the function of synaptic and extrasynaptic NMDA receptors (NMDARs) which mediate Na^+^ and Ca^2+^ influx into neurons and regulate membrane polarization [82].

### 2.3. Tau Protein in the Nucleus of Neurons

Tau is localized in the nucleus of neurons, more precisely, in nucleolar organized regions of the nucleolus [83,84,85,86]. Since nucleolus is the center of ribosomal RNA (rRNA) synthesis and processing, several studies which described localization of tau in parts of nucleolus suggest that tau can be involved in rRNA-coding DNA transcription and rRNA processing [86,87]. Tau can directly bind DNA [88] and RNA [89] and protects them from oxidative damage [90,91] which helps to maintain DNA and RNA integrity [92]. Furthermore, tau-DNA interaction is modulated by tau phosphorylation, which strongly reduces the ability of tau to bind DNA [93]. Hyperphosphorylation, which is one of the major hallmarks of the pathological forms of tau, may also influence its nucleocytoplasmic transport. According to the recent study, hyperphosphorylated tau directly interacts with a subunit of nuclear pore complex—nucleoporin NUP98 causes its mislocalization and disrupts the nucleocytoplasmic transport [94].

### 2.4. Big Tau

**Figure 3:**
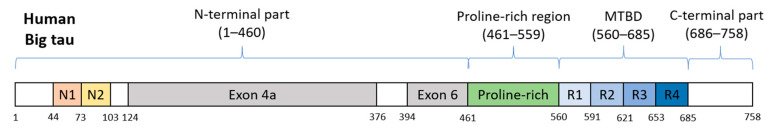
Schematic representation of human Big tau. Its counterparts in rat and mouse possess 752 or 733 aa and share 74.9% or 74.2% identity, respectively. Exons 4a and 6 are responsible for differentiation from the brain tau protein.

Big tau was initially known from studies on rats and mice where tau proteins were detected in their tissues, as well as, in cell lines derived from these species. This high-molecular-weight form of a rat and murine tau has an apparent molecular weight of ~110 kDa and possesses 733 or 752 amino acids (aa), respectively [95,96], as a result of the involvement of exon 4a and alternatively spliced exon 6 of *MAPT* gene in its transcript [97,98]. It was shown that Big tau is expressed only in the peripheral nervous system (PNS), neurons of the optic nerve, but also in specific CNS neurons with long axons projecting to the periphery [96,99]. In human sequence databases, Big tau is also designated as the PNS tau. Human Big tau (Figure 3) has not been confirmed experimentally in human tissues or human-derived cell lines so far, and its specific function remains unanswered. In light of the known functions of brain tau protein, several benefits of Big tau due to its increased length were proposed. The most important is the increased spacing between microtubules observed in processes of Sf9 cells overexpressing Big tau, which may reduce the energy required for axonal transport [100]. Furthermore, the elongated N-terminus of Big tau was proposed to reduce the rate of phosphorylation of motor proteins, and not mitigating their activity, thus supporting uninterrupted axonal transport [97,101].

### 2.5. Extracellular Tau Protein

Mounting evidence shows that pathological forms of tau protein spread from diseased to healthy cells and transform physiological tau to its misfolded pathological forms which in turn self-aggregate and form PHF and NFT [102,103]. It was shown that neuronal activity, accompanied by synaptic transmission, mediates tau protein release into extracellular space, mainly in an exosome-bound form, but also in a soluble form, and that this process occurs under both physiological and pathological conditions [63,64,65,66]. One of the suggested pathways of pathological tau spreading is connected to the resident macrophages of CNS–microglia [73]. Microglia have both phagocytic and secretion properties and can play a key role in the spreading of tau pathology [104]. Microglial exosomes serve as a medium for intercellular transport of cytokines, miRNAs, and other regulating factors [105]. The fact that microglia can be involved in the spreading of pathological tau forms is supported by several experiments. The group of Asai showed that depletion of the microglia in mouse brain significantly slowed propagation of tau between cells and that tau spreading was mediated by microglial exosomes [106]. Another research group demonstrated that reactive, inflammatory microglia can contribute to the spreading of tau pathology [73].

## 3. Tau Interaction Partners (TIPs), Their Biological Functions and Related Molecular Pathways

The understanding of tau function and behavior is mainly based on genetic experiments such as in-site mutagenesis, overexpression, or depletion of tau protein in animal or cellular models [107,108,109]. Moreover, studying physical protein-protein interactions [PPIs] significantly broadened the knowledge about tau protein function and its relationship to various physiological processes [7]. Since PPIs are elementary for many processes and it is supposed that their dysfunction or deregulation is located upstream of various pathological conditions, it is important to understand the tau protein interactome and dynamics [1].

Studies that focused on tau protein forms present in PHF and NFT showed that tau present in pathological lesions can be phosphorylated, truncated, glycosylated, nitrated, and ubiquitinated [110,111,112,113,114,115,116,117]. Therefore, it is likely that proteins involved in molecular pathways connected to the above-mentioned posttranslational modifications can have an impact on the pathological processes in tauopathies. The proteins and other compounds identified in NFT are listed in Table 1. Their presence in NFT could be the consequence of their physiological or pathological interactions with tau [118,119,120]. However, their presence in NFT can also result from a damaged fine-balanced chaperone system, altered nature of individual proteins due to pathological conditions (oxidation, modifications, etc.), or nucleocytoplasmic coagulation of proteins [121].

Physiological IPs play different roles in cells, and they are a part of various molecular pathways like energy metabolism, chaperone complex, apoptosis, organization of the cytoskeleton, and signaling pathways. Under pathological conditions, abnormal interactions of tau with its partners may occur as a consequence of the deregulation of any molecular pathway. Vice versa, newly emerging pathological forms of tau proteins within the cell can deregulate or block physiological processes in the cell and interact with proteins that do not occur under physiological conditions.

**Figure 4:**
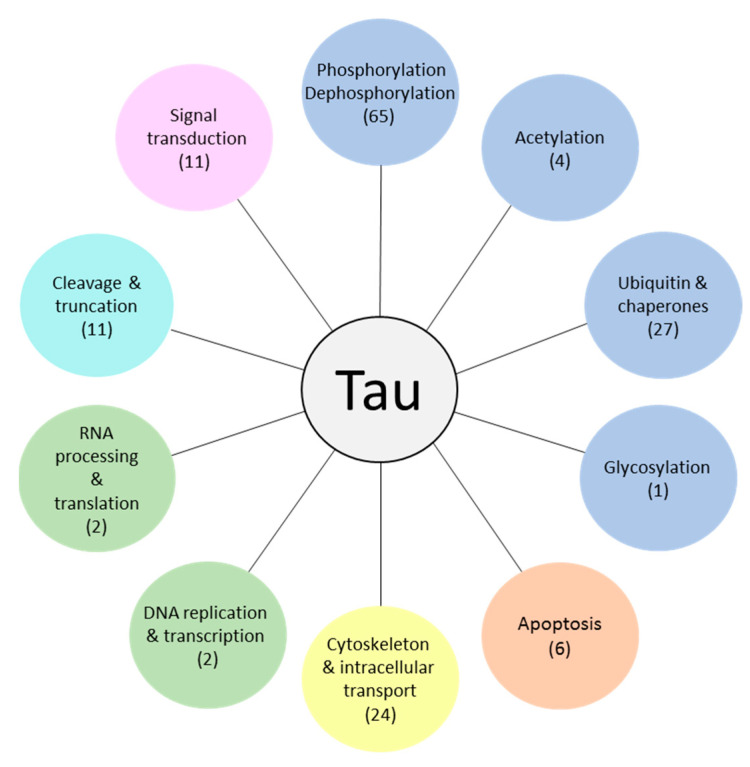
Overview of validated TIPs distributed according to their function. In brackets: the number of validated TIPs for the particular group.

PPIs are summarized in several databases, however, all of them contain many hypothetical, predicted, or experimentally non-validated interactions. The following PPI databases are the best known (with the number of *Homo Sapiens* interactions in brackets): BioGRID (789073) [149], GPS-Prot (395501) [150], DIP (9141) [151], IntAct (703717) [152], MINT (10143) [153], STRING (12628534) [154] and IID (1099176) [155]. We reviewed all of them and found that the BioGRID (Biological General Repository for Interaction Datasets) was the most representative regarding TIPs (reporting up to date 245 TIPs). After a detailed review of all listed studies reporting the interactions, we excluded the non-validated interaction partners, which were derived mainly from large-scale interaction studies with no additional validation experiments. We then examined the resulting 153 experimentally validated TIPs by reviewing the scientific literature and protein databases. TIPs can be divided into several groups according to their molecular functions and their impact on tau protein physiology and pathology (Figure 4).

### 3.1. Tau as a Substrate for Kinases and Phosphatases

Phosphorylation and dephosphorylation of tau protein are physiological processes that regulate the binding of tau to microtubules. Increased phosphorylation of tau MTBD causes the release of tau from microtubules [116,156,157,158,159]. Under pathological conditions, tau protein is hyperphosphorylated, unable to bind microtubules, and subsequently aggregates and forms PHF and NFT. In AD, several pathological phosphorylations were identified [117,160,161,162,163,164], and the overall phosphorylation of tau is elevated by approximately four-fold in comparison to a healthy brain. This ratio may be even higher due to post-mortem delay of analyzed brain samples where tau dephosphorylation by phosphatases occurs [116,165]. The longest isoform of human tau protein has 85 potential phosphorylation sites of which 71 were experimentally documented [160,163,166,167,168]. This represents 19.3% of tau amino acids available for phosphorylation/dephosphorylation.

Among the known, and validated TIPs, 65 of them are involved in phosphorylation and/or dephosphorylation. Fifty-four of them are protein kinases (Table 2), seven phosphatases (Table 3), and four important kinase or phosphatase modulating proteins (Table 4). The protein kinases, which represent the majority of TIPs, were extensively reviewed by Martin et al. [169] and partially by Guo et al. [18]. The phosphatases, including PP2A, which contributes to approximately 70% of brain dephosphorylation activity [170], were deeply reviewed in Martin et al. [171] and by Guo et al. [18].

### 3.2. Tau-Interacting Partners Involved in Acetylation and Deacetylation of Proteins

Acetylation affects around 80–90% of all translated human proteins [241]. Acetylases are enzymes with acetyltransferase and deacetylase activity, and their role is the addition or removal of an acetyl group to the N-terminal or lysine residues of proteins. Acetylation of proteins regulates many processes, for example, transcription and memory consolidation by histone acetylation [242,243], localization in the cell [244], modulation of PPIs, and others [245]. Acetylation of tau protein on the Lys^280^, Lys^281^, and Lys^311^ residues impairs tau binding to microtubules [246,247], which leads to increased pools of cytosolic tau available for pathological aggregation. Tau itself possesses intrinsic acetyltransferase activity that allows tau self-acetylation [248]. Tau acetylation occurs before tau fibrillization into PHF suggesting that it could be an upstream pathological event. Besides AD, acetylated tau at the Lys^280^ is also present in PHF from patients with corticobasal degeneration [247]. In AD, deregulation of acetylation of both nuclear and cytoplasmic non-histone proteins occurs. Acetylated-tau is present through all of the stages of AD, peaking in the end stages of the disease [249]. Moreover, acetylation of tau inhibits its degradation, and along with increasing the concentration of unbound tau, it contributes to tau aggregation and pathology [250].

Currently, four proteins acetylases are validated as TIPs: CREB-binding protein (CBP) [251], histone acetyltransferase p300 (p300 HAT) [246,250,252], NAD-dependent protein deacetylase sirtuin-1 (SIRT1) [250] and histone deacetylase 6 (HDAC6) [253].

CBP and p300 HAT are highly homologous enzymes, which regulate transcription via chromatin remodeling and also acetylate non-histone proteins [247,254,255]. Both enzymes can acetylate up to 19 sites in tau protein located in the PRR and MTBD. The loss or over-expression of CBP and p300 HAT is responsible for neuronal death, which suggests that only the balanced and specific activity of these acetyltransferases is neuroprotective [256].

HDAC6 is a deacetylase mainly found in the cytoplasm, but also in smaller amounts in the nucleus [257]. Its main function is the deacetylation of several cytoplasmic proteins. Under pathological conditions, the HDAC6 is overexpressed, along with increased translocation to the nucleus, resulting in a decreased levels of brain-derived neurotrophic factor, a critical factor for synaptic repair and plasticity, leading to synaptic loss [258]. The overexpression of HDAC6 in AD can result in lower levels of acetylated tubulin [253], and thus impairment of transport, or increase in oxidative stress by deacetylation of peroxyredoxins resulting in lowering their ability to eliminate oxidative response products [259]. Furthermore, overexpression of HDAC6 elevates the burden of tau [260], which might also play a role in neuroinflammation [261,262]. HDAC6 can also deacetylate lysine residues in MTBD of tau which enhances tau phosphorylation, and aggregation [252].

The deacetylase SIRT1 links transcriptional regulation to the energy homeostasis of the cell. It plays a role in different processes like cell cycle, response to DNA damage, metabolism, apoptosis, and autophagy [263,264]. The expression of SIRT1 is decreased in aged neurons, and under neuropathological conditions [265,266]. SIRT1 deacetylates lysine residues in the PRR of tau protein [250] and has a neuroprotective role during neuronal injury and neurodegeneration [267]. Deletion of the *Sirt1* gene in the mouse model of tau exacerbated their mortality, synapse loss, and cognition deficits. After induced expression of *Sirt1* in the same model, the spread of tau pathology in the brain was attenuated [268]. In AD, SIRT1 levels are decreased and negatively correlated with the accumulation of pathological tau acetylated at Lys^174^ [265]. Deficiency of SIRT1 leads to synapse loss, impaired memory and spatial learning [269], elevated levels of proinflammatory cytokines, and accumulation of hyperphosphorylated tau due to low tau turnover [250]. Thus, the interaction of SIRT1 with tau protein has neuroprotective effects.

### 3.3. Tau-Interacting Partners Involved in Glycosylation

Only one protein from this group was experimentally validated for interaction with tau: the *O*-linked *N*-acetylglucosamine transferase (OGT) [270]. OGT is a glycosyltransferase that catalyzes the addition of a β-*N*-acetylglucosamine (GlcNAc) moiety to threonine or serine residues via an *O*-glycosidic linkage [271]. In a healthy brain, the tau phosphorylation sites are protected by *O*-GlcNAc modification. Thus, a competitive modification of Ser and Thr residues between *O*-GlcNAcylation and phosphorylation occurs. In vitro glycosylation of tau at Ser^356^ slowed tau aggregation [272]. In AD, impaired glucose metabolism leads to the reduction of UDP-GlcNAc, thereby decrease in *O*-GlcNAc levels, thus facilitating the tau phosphorylation and aggregation [273,274]. After the phosphorylation of tau, the chance of tau being *O*-GlcNAcylated is lower [275].

### 3.4. Interactions of Tau with Ubiquitin-Proteasome System and Chaperone System

Two major degradation pathways are known: the ubiquitin-proteasome and the lysosomal pathway, and these pathways are tightly regulated with molecular chaperones [276]. It is proposed that a fine-balanced chaperone system, which participates in correct protein folding and degradation of misfolded proteins could play an important role in the accumulation of disordered toxic tau species. Indeed, it has been shown that the chaperone system is impaired in tau pathology [277]. However, it is unclear whether this event is upstream or downstream of tau pathology.

#### 3.4.1. Tau-Interacting Proteins Involved in the Ubiquitin-Proteasome Pathway

The ubiquitin-proteasome pathway of protein degradation involves the attachment of ubiquitin moieties to proteins which ensures their targeting to proteasomal degradation [278]. The process of ubiquitination starts by activation of ubiquitin by ubiquitin-activating enzymes (E1). Subsequently, the activated ubiquitin is transferred to a ubiquitin-conjugating enzyme (E2) and then attached to the target protein by ubiquitin ligases (E3) [279]. Some of the experimentally detected E2 ubiquitin-conjugating enzymes (UBE) interacting and ubiquitinating tau protein are UBE2D2 [280], UBE2D3 [281], and UBE2W [280,281,282,283]. The UBE2W is likely specific to intrinsically disordered proteins, such as tau, because of its partly disordered and flexible C-terminal domain. This flexibility allows recognition of disordered N-terminal domains of proteins and promotes ubiquitination [284].

One of the E3 ubiquitin-protein ligases that interact and modify tau proteins is parkin [285,286]. Parkin regulates mitochondrial trafficking, mitophagy, endosomal sorting, synaptic transmission, programmed necrosis, ER stress, inflammation, and cellular homeostasis [287,288,289,290]. Despite its various important cellular processes, there is not enough evidence about its direct involvement in tau pathology.

Other E3 ubiquitin-protein ligases reported to interact with tau are CHIP [281,291], axotrophin [292], and TRAF6 [293]. The CHIP ligase targets misfolded chaperone substrates to proteasomal degradation [294], and along with HSP70 chaperones, facilitates ubiquitination and degradation of tau protein which enhances cell survival [280]. Studies show that CHIP overexpression can promote tau aggregation [285], and ubiquitination of tau by axotrophin significantly reduces the affinity of tau protein to microtubules [292].

E3 ubiquitin-protein ligase TRAF6 acts in cooperation with Sequestosome-1 (SQSTM1) via complex formation. SQSTM1 is a multifunctional TIP [293,295] that regulates the elongation of ubiquitin chains on the surface of substrates and intensifies their targeting signal [296,297,298]. The SQSTM1 is essential for the shuttling of tau to proteasomal degradation because TRAF6 alone is not capable to polyubiquitinate tau [293]. Furthermore, SQSTM1 also functions as a receptor for selective macroautophagy of polyubiquitinated proteins and aggregates [299,300].

The neddylation is a process similar to ubiquitination; however, instead of ubiquitin, the NEDD8 is attached to proteins [301]. NEDD8 by itself and its protein conjugates are targeted to proteasomal degradation by NEDD8 ultimate buster 1 (NUB1), which was also identified as a tau-interacting protein [302,303]. The work of Richet et al. [303] showed that in SK-N-SH cells, NUB1 disrupts the interaction of tau with GSK3β kinase thus lowering pathological phosphorylation and aggregation of tau. The same group also recently showed that SQSTM1 specifically interacts with NUB1. Using the SH-SY5Y neuroblastoma cell model, it was demonstrated that NUB1 reduced the levels of insoluble tau aggregates. NUB1 also increased the autophagy-lysosomal pathway which facilitated the release of tau from SH-SY5Y cells [304]. These data suggest that NUB1 enhances the viability of diseased neurons at the expense of facilitating tau spreading between cells.

#### 3.4.2. Tau-Interacting Proteins Involved in Chaperone System

The chaperone system is composed mainly of heat shock proteins (HSPs), which are involved during stress conditions. These proteins mainly function as molecular chaperones and regulate several diverse cellular processes, including protein folding, targeting, transport, degradation, and signal transduction. Under stress conditions, they assist in protein refolding and suppress aggregation, which promotes the maintenance of cellular homeostasis. HSPs are classified into the following families according to their molecular size: HSP90, HSP70, HSP60, HSP40, and small HSPs.

HSP70 chaperones assist in the stabilization and folding of many substrates and are found in most cellular compartments [305]. In humans, 11 genes encoding HSP70 family members have been identified [306]. The four chaperones of this family are known to interact with tau: HSPA1A [307], HSPA4 [307], HSPA5 [308] and HSPA8 [309]. All HSP70 proteins have a conserved N-terminal ATPase domain that binds and hydrolyses ATP and a C-terminal substrate-binding domain. The co-chaperone of HSP70, BAG family molecular chaperone regulator 1 (BAG1), serves as a nucleotide-exchange factor and promotes the release of ADP from HSP70 chaperones [310]. BAG1 was also found to interact with tau; however, only in complex with HSP70. Despite this indirect interaction, BAG1 is an important regulator in tau pathology because of its inhibitory effect on ubiquitin-independent 20S proteasomal degradation [311]. However, HSP70 chaperones are potent inhibitors of tau aggregation by preventing the formation of tau oligomers and PHF. It has been shown that HSP70 chaperones protect neuronal functions against the toxic effects of tau aggregates and oligomers [312].

Another co-chaperone of HSP70s interacting with tau is the DnaJ homolog subfamily A member 1 (DNAJA1). It was demonstrated that the over-expression of DNAJA1 mediated ubiquitin-dependent clearance of tau, while *DNAJA1* knockdown facilitated tau accumulation [313].

The HSP90 heat shock protein family is an essential component of the eukaryotic cytosol where they stabilize misfolded proteins and regulate the activity of various signaling proteins, including steroid hormone receptors, tyrosine kinases, nitric oxide synthase, and calcineurin [314]. Two HSP90 proteins are known to interact with tau: HSP90α [315,316,317] and its co-chaperone and activator AHSA1 [318], which triggers the ATPase activity of HSP90α thus increasing its activity [319]. The interaction of tau with both these proteins is pathological. HSP90 and its activator AHSA1 can mediate tau oligomerization and aggregation [320,321,322].

Heat shock protein β-1 (HSPB1), which belongs to the family of small HSPs, preferentially interacts with hyperphosphorylated tau in the human brain [323]. In the cell model, it decreased hyperphosphorylated tau levels, increased the abundance of dephosphorylated tau, and suppressed tau-mediated cell death [324].

The Clusterin (CLU) is a chaperone that prevents the aggregation of misfolded proteins [325]. It is present in two main forms: the secreted (sCLU) and intracellular (iCLU). Total levels of both sCLU and iCLU are significantly increased in AD, and these levels are proportional to overall levels of insoluble Aβ and tau aggregates [326]. iCLU was identified as a TIP, which also interacts with another TIP: BIN1 (see Section 3.7). It was shown that expressions of both iCLU and BIN1 were associated with misfolded tau in AD [327]. Expression of certain coding CLU variants linked to AD risk led to increased levels of iCLU. Therefore, the iCLU and BIN1 interaction might impact Tau function in neurons, and could be involved in the etiology of tau pathology in AD.

Protein isomerization is an essential physiological process involved in protein folding and maturation [328]. Two types of isomerization in proteins are known: prolyl *cis-trans* isomerization and disulfide isomerization. The prolyl *cis-trans* isomerization shifts the proline peptide bonds between *cis* and *trans* conformation, thus causing changes in protein secondary structure [329]. The 2N4R tau contains 43 proline residues, the majority of which are located in its PRR. Thus, isomerization of the proline peptide bonds between *cis-trans* conformation can influence tau protein structure and behavior [330,331]. Moreover, the proline *cis-trans* isomerization also regulates tau protein phosphorylation. Deregulation of this process causes phosphorylation of tau protein at AD-specific phospho-sites [332].

Five peptidyl-prolyl *cis-trans* isomerases: FKBP1A [315], FKBP4 [333], FKBP5 [318], PIN1 [334], cyclophilin D (CypD), and one protein disulfide-isomerase (PDI), are known to interact with tau. PDI is responsible for proper protein folding by both enzymatic and chaperone activity. It catalyzes the rearrangement of the formed disulfide bonds to correct positions [335]. It was shown that tau aggregation is significantly prevented by the binding of PDI to monomeric tau proteins [336]. Furthermore, the PDI strongly inhibits the tau seeding process which precedes tau aggregation [337,338]. In AD, PDI is S-nitrosylated which causes inhibition of this enzyme [339,340].

The peptidyl-prolyl *cis-trans* isomerases play an important role in protein folding through the isomerization of proline peptide bonds between *cis* and *trans* conformation. Their functional effects on tau protein and related pathology were extensively discussed in reviews of Blair et al. [341] and Peak et al. [342]. They play different roles in tau pathology, they can either facilitate tau aggregation, like FKBP4 and FKBP5, or can be neuroprotective, like PIN1 or FKBP12.

Cyclophilin D (CypD) [343], besides its peptidyl-prolyl activity, also participates as a regulator of the mitochondrial permeability transition pore in mitochondria, which is responsible for the Ca^2+^ release [344,345,346]. Mitochondrial damage and Ca^2+^ imbalance are pathological features of AD [347,348,349]. It was suggested that increased expression of CypD could play an important role in the neurodegenerative process as in AD. It was shown that Aβ interacts with CypD [350,351], and induces mitochondrial and neuronal stress. Recent studies also demonstrated a link between mitochondrial dysfunction and tau pathology as a contributor to AD [352,353].

### 3.5. Interactions of Tau with Proteins Regulating Programmed Cell Death

The apoptosis, one of the consequences of the developed Aβ and tau pathology [354], is an important physiological cell process mediated by various proteins including caspases, the cysteine aspartyl proteases [355]. Interactions of tau with caspases were confirmed by the study of Gamblin et al., where the authors suggest that caspases involved in Aβ-induced neuronal apoptosis could contribute to pathological cleavage of tau. They showed that tau is cleaved at Asp^421^ in vitro by caspases-1,-3,-6,-7, and -8, and the same cleavage product of tau is produced in primary rat cortical neurons after treatment with fibrillar Aβ [112]. This cleavage of tau with caspases generates pathological truncated tau species which can aggregate, lead to neurofibrillary pathology, and contribute to neuronal death [356,357]. Although the caspase activation precedes the formation of neurofibrillary tangles, the soluble tau species are the caspase activators that augments tau truncation, thus contributing to NFT formation [45,358].

Caspase-1 is activated by proteolytic cleavage by other caspases, and it is a part of a pathway called pyroptosis: a lytic and inflammatory form of programmed cell death [359].

Caspase-3 is involved in the activation cascade responsible for the execution of apoptosis. When caspase-3 is activated, it cleaves its substrates caspase-6, -7, and -9 which simultaneously activates them. In the AD brain, caspase-cleaved tau colocalizes with both intracellular Aβ and activated caspase-3 [113]. Through the cleavage of Ser/Thr Kinase 1 (Akt), caspase-3 regulates tau phosphorylation via the GSK3β kinase pathway [360]. Furthermore, upregulation of active caspase-3 led to the accumulation of caspase-3-cleaved tau in the traumatic brain injury model [361]. These data suggest that abnormal activation of caspase-3 may lead to progressive tau pathology.

Caspase-6 cleaves tau at three different sites in comparison to other caspases: Asp^13^ [362], Asp^402^ [363] and Asp^421^ [364]. Cleaved tau colocalized with active caspase-6 within NFTs in AD. Furthermore, active caspase-6 was identified in the mild stage of AD which supports its role in the early stages of tau pathology [365,366]. Caspase-6 (along with caspases-3 and -8) cleaves amyloid precursor protein (APP) at Asp^664^ releasing C-terminal p31 cytotoxic fragment which can induce apoptotic pathway cascade [367,368,369,370].

Caspase-7 is the effector caspase in programmed cell death which induces the Gasdermin-D-independent pore formation [371]. It cleaves tau at Asp^421^ [113]. In the study by Ayers et al., the individuals homozygous for AD-risk *APOE4* allele and had loss-of-function mutation in the *CASP7* gene did not develop the AD [372], this implicates the neurotoxic role of caspase-7. However, a genetic association study identified a different, rare missense variant of the *CASP7* gene to be robustly associated with familial late-onset AD [373].

Caspase-8 is the most upstream protease in the activation cascade of caspases [374]. It activates caspase-3 by proteolytic cleavage [375]. Furthermore, it has been shown that in the brain of AD patients, the active form of caspase-8 is abundantly present in NFT-bearing neurons [376]. In contrast, a gene association study that found two mutations in the *CASP8* gene: K148R and I298V also showed in vitro that mutation I298V had an attenuating effect on caspase-8 activity [377]. Interestingly, de Calignon et al. noted that after the formation of NFT in neurons, the caspase activity is suppressed [45].

The next TIP involved in apoptosis is the neuronal pentraxin-1 (NPTX1) [77]. Under physiological conditions, the NPTX1 is released from synapses into the synaptic cleft, binds extracellularly to AMPA glutamate receptors (AMPARs), and stabilizes them on dendritic surfaces [378,379]. Thus, the secreted NPTX1 binds and recruits AMPARs, promoting the formation of an active synapse [380,381]. It has been shown that under potassium deprivation and reduced neuronal activity, NPTX1 protein is overexpressed along with other proteins involved in programmed cell death [382,383]. Furthermore, exposure to Aβ also increased NPTX1 expression leading to reduced neurite outgrowth and increased apoptosis. Moreover, NPTX1 overexpression alone reproduces the effects of Aβ on neurite damage and apoptosis [384].

### 3.6. Proteolytic Cleavage and Truncation of Tau Protein

Protein degradation is an important physiological process, which is responsible for the removal of aged, damaged, or misfolded proteins with the possibility of their constituents being recycled in the process [385]. Proteolytic cleavage of specific proteins is the common denominator of many neurodegenerative diseases including huntingtin protein in Huntington’s disease [386,387], α-synuclein in Parkinson’s disease and Lewy body dementia [388], ataxins in cerebral ataxia [389,390], prion protein in prionosis [391,392] and tau protein in Alzheimer’s disease, as mentioned in Section 1 [117]. Experiments deciphering PHF showed that the “minimal protease-resistant” core of paired helical filaments are mainly composed of tau proteins and that the majority of them are truncated [393,394,395]. A general consequence of proteolysis is the production of various fragments with a toxic gain-of-function that can be translocated into an inappropriate cell compartment [358,396]. Pathological tau protein fragments can either switch on the cell death cascade or induce and drive protein aggregation. Mounting evidence supports the idea that truncated protein fragments are upstream in the pathological cascade and can form the initial seeds for the aggregation in neurodegenerative diseases [45,397,398,399]. For example, the cleavage of tau monomers to truncated fragments supports its mounting into oligomers and PHF [117].

Two calcium-regulated thiol-proteases are known to interact with tau: calpain-1 [400] and calpain-2 [401], and they are major isoforms of calpains in the brain. Calpains can cleave tau in vitro [402]. Calpain-1 participates in long-term potentiation and acts neuroprotective on neurons. On the other hand, calpain-2 activation limits neuronal potentiation leads to silencing of neuronal activity, and neuronal death [403]. Calpain-1 showed an increase in activity in AD from Braak stage III to the late stages of the disease [404]. Activation of both calpains leads to the production of 10.7-kDa tau fragment in neurons (tau 125∓230, related to 2N4R tau), which is neurotoxic [405,406]. Additionally, calpain-1 cleaves tau at Arg^242,^ and this fragment is observed in brains with tauopathy [407]. Furthermore, a recent study by Cicognola et al. showed that in tauopathy, calpain-2 cleaves tau at Lys^224^ [408], a fragment enriched in CSF tau pool in pathological conditions [409].

In addition to thiol-proteases, legumain [410,411] and ubiquitin thioesterase OTUB1 [412] are also known as TIPs. The legumain is predominantly localized in lysosomes and it is a multifunctional enzyme that can have endopeptidase, carboxypeptidase, or ligase activity depending on their milieu [413]. Increased levels of the active legumain along with a higher amount of proteolytically truncated tau were found in the cytoplasm of neurons in AD [410,414]. Ubiquitin thioesterase OTUB1 belongs to the deubiquitination family proteins. It is involved in the processing of poly-ubiquitin precursors, as well as ubiquitinated proteins [415]. OTUB1 was shown to be involved in the deubiquitination of tau protein thus preventing its degradation [416]. Furthermore, expression of OTUB1 in primary neurons increased tau levels, enhanced tau aggregation, and contributed to tau pathology [412].

Cathepsin D (CTSD) is the lysosomal aspartic acid protease involved in cleavage and activation of ADAM30 which leads to amyloid precursor protein (APP) degradation and thus preventing the formation of Aβ peptides and plaque load [417]. The study of Khurana et al., examined the relationship between CTSD and tau in vivo using the *Drosophila* model of AD and showed that CTSD is upregulated with age and has a neuroprotective effect. The deletion of the *Ctsd* gene in this model increased the generation of Asp^421^-cleaved tau protein and exacerbated tau toxicity. Additionally, the authors showed that ablation or silencing of the *Ctsd* gene also in mice and sheep resulted in the truncation of tau at Asp^421^ and caspase-3 activation [418]. CTSD has many cellular functions including activation of enzymes, such as ADAM30, and various enzymatic precursors [419], degradation of intracellular proteins [420,421], activation and degradation of hormones and growth factors [422,423], processing of enzyme activators and inhibitors [424], processing of brain-resident proteins, such as tau [425], myelin [426] or Aβ [427], and regulation of apoptosis [428]. It was shown that genetic variation in the *CTSD* gene is a risk factor for AD [429].

Human high-temperature requirement serine protease A1 (HTRA1) and thrombin are two serine proteases known to cleave tau protein [400]. HTRA1 is able to degrade tau aggregates and fibrils, and patients with elevated expression of HTRA1 had lower amounts of accumulated tau protein in the brain [400,430]. Thrombin, an extracellular protease, cleaves tau protein at various arginine and lysine residues. Its proteolytic activity is inhibited by the phosphorylation of its substrates. PHFs isolated from the AD brain were more resistant to thrombin cleavage than those that were dephosphorylated [431].

The 26S proteasome complex, which comprises 19S regulatory particle (19S RP) and 20S core particle (20S CP) [432], interacts directly with tau protein [433]. Furthermore, two of nine subunits of the base complex of 19S RP are TIPs: the 26S proteasome regulating subunit 7 (PSMC2) [293] and 26S proteasome non-ATPase regulatory subunit 2 (PSMD2) [77]. The main role of 19S RP is the degradation of ubiquitinated proteins in an ATP-dependent manner [434,435,436]. The PSMC2 subunit is a motor protein with ATPase activity. The PSMD2 recognizes and binds ubiquitin bound Usp14, a ubiquitin-specific protease, which cleaves polyubiquitin chains from substrates before entering the 26S proteasome core [437,438]. However, in tauopathies, the ubiquitin-dependent proteasomal system is impaired [75] and tau protein aggregates may inhibit proteasome function [439,440]. Furthermore, pathological protein aggregates may have a suppressive effect on the proteasome system, as demonstrated by Thibaudeau et al. who showed that Aβ oligomers can bind to the 20S CP [432] and thus impair substrate entry into the 26S proteasome [441].

Only one metalloprotease that can degrade tau has been reported so far, the puromycin-sensitive aminopeptidase (PSA) [442]. It was shown that PSA more efficiently degraded soluble tau from the normal human brain when compared to soluble or PHF tau purified from AD brain, very likely due to post-translational modifications and/or aggregation of tau. PSA is upregulated in patients with tauopathies [443], but its role in tau pathology is not known yet.

Presenilin-1 (PSEN1) is a part of the γ-secretase complex, which cleaves integral membrane proteins such as Notch receptors and APP [444,445]. Mutations in this gene are risk factors for developing familial Alzheimer’s disease [446,447]. It has been shown that PSEN1 facilitates the phosphorylation of tau protein through direct interaction with tau and glycogen synthase kinase 3β (GSK3β) and thus guiding the GSK3β into close proximity with tau for phosphorylation [448]. Moreover, two AD-characteristic mutations in PSEN1 increased its binding to GSK3β and enhanced the phosphorylation of tau protein [449]. However, the exact mechanism as to how PSEN1 contributes to tau pathology remains unanswered.

### 3.7. Proteins Involved in the Regulation of the Cytoskeleton and Intracellular Transport

Cytoskeleton regulating proteins are essential for maintaining and regulating the shape and function of the cytoskeleton, axons, dendrites, and synapses [450]. Furthermore, they are important for axonal growth, cellular transport, and axonal signal transmission [451,452]. The cytoskeleton proteins show abnormalities during tauopathies, like the inhibition of assembly or deformation of microtubules and invaginations of the nuclear membrane [453,454]. Cytoskeleton constituents, such as tubulins (4 isoforms) [292,455,456,457] and actin (3 isoforms) [80], which polymerize into filaments and microtubules (MTs) are stabilized and co-organized by microtubule-associated proteins (MAPs) or actin-binding proteins (ABPs) [81].

Another cytoskeleton-regulating TIP is the microtubule-associated protein 2 (MAP2) [458], which belongs to the category of microtubule-binding proteins. Strong MAP2 immunoreactivity was observed mainly in dendrites of neurons and occasionally in neuronal soma. MAP2 regulates the spacing between MTs in dendrites and is involved in dendrite arborization and growth [459]. The induction of long-term potentiation in cultured primary rat neurons and mice hippocampal slices caused translocation of MAP2 from dendritic shafts to dendritic spines and heads. This suggests that MAP2 is involved in processes of synaptic plasticity and learning [460]. The other function of MAP2 is the regulation of axonal transport. It was observed that in sensory neurons MAP2 coordinates the functions of molecular motors kinesin-1 and kinesin-3, where it inhibits slow kinesin-1 and thus allowing fast kinesin-3 to drive cargo transport from the soma into the axon [461]. The importance of physiological interaction of tau with MAP2 has not been revealed yet, however, it was shown that hyperphosphorylated tau protein isolated from AD brain inhibits MAP2-promoted MT assembly [458].

The microtubule dynamics rely on the assembly or disintegration of tubulins on plus-ends of microtubules [462]. These dynamics are regulated by the presence or absence of factors and proteins such as the GTP-bound tubulin dimers [463], tau protein [464,465], or plus-end tracking proteins [466]. The two plus-end tracking proteins are known to directly interact with tau, the microtubule-associated protein RP/EB family member 1 (MAPRE1) [467] and 3 (MAPRE3) [467]. They promote and regulate MT nucleation and elongation [468,469]. Moreover, the MAPRE1 and MAPRE3 also regulate the minus-end of MTs and mediate the tethering of MTs to the Golgi apparatus [469]. Tau protein, through interaction with MAPRE1 and MAPRE3, inhibits their binding to MTs and this inhibition is abolished by tau phosphorylation at Ser^262^ [470]. Furthermore, MAPRE1 and MAPRE3 could be directly involved in tau secretion from the cells [471]. These data suggest the existence of a tight relationship between tau and end-binding proteins.

The next TIP from the class of cytoskeleton-regulating proteins is amphiphysin II [327,472,473], which controls the plasma membrane curvature, shaping, and remodeling [474]. Amphiphysin II is abundantly expressed in the brain and muscle cells [475]. Using genome-wide association studies, the variations in its gene (*BIN1*) were identified as the second most risky genetic factor for sporadic Alzheimer’s disease [476]. It was shown that under pathological conditions amphiphysin II expression is elevated [473,477]. However, the direct function of amphiphysin II in tau pathology remains unknown.

Protein kinase C and casein kinase substrate in neurons 1 (PACSIN1) is a flexible adaptor protein that contains important SH3 and F-BAR domains which are known to interact with many proteins. The F-BAR domain regulates membrane deformation and shaping of the neuronal plasma membrane [478,479]. PACSIN1 interacts with the proline-rich region (PRR) of tau through its SH3 domain, and by this interaction, it coordinates the remodeling of the MT cytoskeleton [79]. Moreover, PACSIN1 plays a role in the reorganization of the actin cytoskeleton thus facilitating the membrane fission during endocytosis [480]. It plays a role in neurodevelopment and during this process, it is upregulated [481]. The PACSIN1 is the key regulator of endocytic removal of developmental NMDARs and their replacement with mature NMDARs [482], which comprise different subunits, and thus possess other synaptic attachment and kinetic properties [483,484,485]. Through this replacement of developmental/mature NMDARs, PACSIN1 regulates the formation of new synaptic connections and is important for learning and memory creation.

One of the novel TIPs that we have recently identified is the brain-specific angiogenesis inhibitor 1-associated protein 2 (BAIAP2) [77]. It is the adapter protein that links membrane-bound small G-proteins to cytoplasmic effector proteins. The BAIAP2 binds and deforms the membranes by its I-BAR domain and may be involved in the formation of membrane curvatures, which are present for example in synapses or dendrites [76,78]. I-BAR domain is also capable of binding and bundling the actin filaments [486]. The SH3 domain of BAIAP2 binds various effectors, whereby many of them are actin modulatory proteins that participate in the nucleation of branched actin filament networks [487,488]. Furthermore, the BAIAP2 participates in the outgrowth of neuronal processes [489] and may be involved in insulin-mediated neurite development and synaptic plasticity [490,491]. BAIAP2 in CNS is located mainly in axonal synapses and dendritic spines and regulates their morphology, as was revealed by electron microscopy [492]. Moreover, their association with psychiatric disorders has been revealed, such as schizophrenia [493,494], autism spectrum disorders [495,496], and attention deficit hyperactivity disorder [497].

The intracellular transport, which is tightly dependent on the cytoskeleton, is commonly impaired in AD. One of the tau-associated proteins implicated in intracellular transport is the hook microtubule-tethering protein 3 (HOOK3), which is expressed predominantly in neurons. HOOK3 belongs to a family of cytoplasmic linkers that participate in endosomal transport. The HOOK3 was shown to associate mainly with tau aggregates, and weak interaction with monomeric soluble tau was also reported [498].

The regulators of nucleocytoplasmic transport are also represented among the TIPs by the GTP-binding nuclear protein RAN [77] and by the nuclear pore complex protein NUP98. The RAN belongs to the Ras superfamily [499], and its GTP-bound form (RAN-GTP) is required for mitotic spindle assembly, and thus for cell proliferation [500,501]. Both RAN and NUP98 mediate the transport of proteins and RNA between the nucleus and cytosol through the nuclear pore complex (NPC) [502,503,504]. A study focusing on nucleocytoplasmic transport in AD showed that hyperphosphorylated tau interacts directly with the FG domain of NUP98 and causes the disruption of nucleocytoplasmic transport. Upon interaction with hyperphosphorylated tau, NUP98 becomes mislocalized to the cytoplasm and promotes tau aggregation into NFT. As NUP98 also interacts with RAN and regulates the RAN-GTP/GDP exchange through NPC, mislocalization of NUP98 results in disruption of nucleocytoplasmic equilibrium of RAN, its depletion in the nucleus and nucleocytoplasmic transport failure [94]. The impact of pathological forms of tau protein on RAN function is currently unknown.

Next TIP, the apolipoprotein E (APOE) [505] associates with lipid particles and plays a role in lipoprotein-mediated lipid transport [506,507,508]. APOE has three common isoforms: APOE2, APOE3, and APOE4 [509], with different levels of lipidation and related functions. Individuals possessing the *APOE4* allele have significantly smaller APOE-containing particles compared to individuals without an *APOE4* allele [510]. Carrying the *APOE4* allele is considered a genetic risk factor for AD, with the increase of risk by three-fold for one allele, and lower age of onset for AD [511]. APOE in the healthy brain plays a neuroprotective role, by binding tau and blocking the phosphorylation sites for kinases [512]. However, only APOE3 and APOE2 can bind tau, not APOE4 [505]. A study proposed genetic interaction between APOE and tau in the development of AD, in a way where the polymorphic tau G allele represents an additional risk factor in the individuals carrying the APOE4 allele, with a five-fold increased risk of AD development [513]. In clinical AD, Aβ mediates the association of APOE with PHF, which correlates with cognitive decline [514].

The motor protein kinesin-1 was also identified as a tau-interacting protein. More precisely, its two light chains, the kinesin light chain 1 (KLC1) and 2 (KLC2) were identified as TIPs [60,515]. KLC1 and KLC2 are present in the complex with the kinesin-1 heavy chain and are responsible for the binding of cargo membrane vesicles, which kinesin-1 motor complex transports along the axon from the neuronal soma to synapses. By interacting with tau protein, both KLC1 and KLC2 participate in the axonal transport of tau in healthy neurons [60]. Whereas the KLC1 is enriched in neurons, the KLC2 is ubiquitously expressed across the various tissues [516]. In the healthy brain, the KLC1 also participates in the transport of amyloid precursor protein (APP) through its attachment to the KLC ligand calsyntenin-1 [517,518], a transmembrane protein present in membranes of APP-containing vesicles in the Golgi [519]. The phosphorylation of KLC1 on Ser^460^ reduces the interaction of KLC1 and calsyntenin-1 thus leading to reduced axonal transport [520]. In the AD brain, the levels of the KLC1 are reduced and the phosphorylation of the Ser^460^ is increased which results in the inhibition of axonal transport of APP [521]. Similarly, the levels of the KLC2 are also decreased in the frontal cortex of AD patients [522]. In the animal model expressing pathological tau proteins, a simultaneous reduction of kinesin light chain expression slowed axonal transport and increased the accumulation of hyperphosphorylated tau and tau aggregates [523].

Another TIP is the dynactin subunit 1 (DCTN1) [61]. This protein is part of the dynactin complex which is responsible for recruiting and tethering the dynein motor complex to MTs [524,525]. Interaction of tau with DCTN1 enhances the binding of the dynactin complex to MTs [61]. Direct involvement of DCTN1 in tau pathology has not been revealed yet.

The mitochondrial import receptor subunit TOM20 homolog (TOMM20) is the part of the translocase of the outer mitochondrial membrane (TOM), a protein complex responsible for the recognition and translocation of cytosolically synthetized proteins into the intermembrane space of the mitochondria [526]. The evidence of TOMM20 as TIPs arises from the study by Amadoro et al. which showed that TOMM20 binds caspase-truncated tau [343]. In AD, the reduced immunoreactivity of TOMM20 was observed. The decreased levels of TOMM20 can lead to an impairment of the recognition and binding of proteins intended for translocation into mitochondria [527,528]. This could contribute to the damage of oxidative phosphorylation as seen in AD. Additionally, the TOM complex can bind and translocate Aβ peptides which results in the accumulation of Aβ in mitochondrial cristae [529].

The ADP/ATP translocase 1 (ANT1) is an ADP-ATP antiporter located at the mitochondrial inner membrane and mediates the import of ADP into the mitochondrial matrix for ATP synthesis in exchange for the export of ATP [530]. Its *ANT1* gene is involved in the maintenance and replication of mitochondrial DNA [531], and its mutations are associated with different mitochondrial disorders affecting the brain [532]. Similarly, as in the case of TOMM20, the caspase-truncated tau fragment can bind ANT1, which either alone or in association with other synaptotoxins can induce synapse decay [343]. The interaction of truncated tau with ANT1 also inhibits its ADP/ATP exchange activity which is one of the factors triggering mitochondrial dysfunction.

The excitatory amino acid transporter 2 (EAAT2) was also identified as TIP and was shown to preferentially interact with tau phosphorylated at AD-characteristic phospho-sites. Furthermore, EAAT2 is recruited into NFT which suggests some role for EAAT2 in tau pathology [533].

Alpha-synuclein (α-syn) [534] is mainly distributed in the presynaptic terminals of neurons [535]. In the healthy brain, α-syn has many different functions like modulation of vesicle trafficking [536], the suppression of apoptosis [537], modulation of synaptic plasticity [538], or chaperone activity [539]. In pathological conditions, α-syn aggregates and forms Lewy bodies, which are typical hallmarks of Parkinson´s disease and other synucleinopathies. In around 50% of AD cases, α-syn pathology is also reported [540,541]. It was shown that the α-syn monomers assemble into amyloid-like fibrils which are able to interact with tau, resulting in inhibition of microtubule assembly and their stabilization. The tau subsequently aggregates which could promote the pathological pathway in synergy [542,543]. α-syn also increases the GSK3-mediated phosphorylation of tau that can further enhance the progress of disease [544]. Furthermore, it was proposed that pathological α-syn is able for cell-to-cell transmission, similar to prion diseases [543].

### 3.8. Tau-Interacting Proteins Involved in DNA Replication and Transcription

Neurons are post-mitotic cells, which are unable to further divide, and are in the resting phase of the cell cycle [545]. In tauopathies, like AD, several changes influence the processes of replication and transcription. It was found that translocation of tau to the nucleus, where it binds and protects DNA under physiological conditions, is reduced upon its hyperphosphorylation; thereby, heterochromatin organization is disrupted leading to cell cycle re-entry and neuronal death [546]. Furthermore, dysregulated gene expression and rRNA synthesis occur resulting in the rise of altered protein synthesis [547,548]. From the group of DNA replication and transcription proteins only two are currently verified as TIPs: the apoptosis-antagonizing transcription factor (AATF) [549] and mothers against decapentaplegic homolog 2 (SMAD2) [550].

AATF is a transcriptional regulator involved in cell proliferation. AATF binds the retinoblastoma protein (Rb) and inhibits its function. The role of Rb is growth suppression via expressional regulation of genes required for DNA synthesis and cell progression [551]. AATF is also an inhibitor of Aβ production in cells undergoing apoptosis by binding and blocking the pro-apoptotic WT1 regulator (PAWR) activity in the regulation of APP processing [115]. On the other hand, AATF participates in neurodegeneration due to its capability to stimulate DNA synthesis and induce quiescent cells to re-enter the cell cycle. AATF–tau association is also shown to be modulated during the onset of neuronal apoptosis in the cytoplasm of cerebral granule neurons, and this interaction is progressively lost in apoptosis [549]. However, the consequences of AATF interaction with tau have not been examined so far.

SMAD2 acts in a complex of SMAD2/SMAD2/SMAD4 or SMAD2/SMAD3/SMAD4 as a transcriptional co-activator on SMAD-binding elements of DNA. SMAD2 is a part of the TGFβ signaling pathway, and along with other SMAD proteins, it is the most important effector of this cascade. TGFβ activates the phosphorylation of SMAD2 and SMAD3 and starts the assembly and translocation of the SMAD2/SMAD3 complex into the nucleus [552]. In AD, SMAD2 is also a part of the TGFβ/SMAD2/STAT3 signaling pathway which is activated by APOE, the important risk factor for AD, and increases the amyloidogenic processing of APP leading to Aβ formation and contributes to cognitive decline [553]. Inversely, SMAD2 plays a role in CX3CL1/TGFβ/SMAD2 pathways which control adult neurogenesis [554]. Thus, SMAD2 signaling pathways can contribute either to neurodegeneration or neuroprotection. Furthermore, it was shown that the interaction of hyperphosphorylated tau with phosphorylated SMAD2 resulted in reduced translocation of SMAD2 into the nucleus [555].

### 3.9. Tau-Interacting Proteins Involved in RNA Processing and Translation

The group of RNA processing and translational proteins is a part of the large protein family, called RNA-binding proteins (RBPs), which are active in the processes like mRNA maturation including splicing, targeting, degradation, and translation of the RNA [556,557]. In AD, several changes to RBPs, and thus to the processing and translational machinery occur. The activity of the spliceosome in AD is impaired resulting in a lower splicing efficiency and production of mRNAs with introns. Splicing deficiency in AD leads to an altered expression of protein-encoding genes like APP which can be detrimental [558]. Furthermore, impaired alternative splicing leads to a dendritic loss in primary neurons and weakened memory in mice [559]. Two proteins from the group of RBPs were validated as TIPs: the U1 70 kDa small nuclear ribonucleoprotein (SNRNP70) [560] and the T-cell-restricted intracellular antigen-1 (TIA1) [561].

The SNRNP70 is a component of the U1 small nuclear ribonucleoprotein (snRNP) complex which is responsible for the recognition of the pre-mRNA 5’ splice-site and the subsequent assembly of the spliceosome [562]. In AD, SNRNP70 is internally cleaved, and its 40 kDa fragment participates in the formation of protein aggregates and was found in close proximity with NFTs [558]. Moreover, this fragment exerts toxic effects on neurons [563]. The truncated, aggregated forms of SNRNP70 are able to bind and sequester the soluble SNRNP70 into insoluble aggregates [564]. The SNRNP70 aggregation occurs earlier in AD and its levels correlate more with Aβ plaques compared to pathological tau aggregates. It was shown that in AD, the LC1/BAD domain of SNRNP70 interacts with tau, a process that does not occur under physiological conditions [560], and it causes mislocalization of SNRNP70 from the nucleus to the cytoplasmic phospho-tau aggregates. These pathological changes of SNRNP70 affect the whole U1 snRNP complex, leading to the loss of the spliceosome function and thus impaired splicing of pre-mRNAs [558,565].

TIA1 is a protein with several different functions in neurons. It plays a role in the regulation of RNA localization and utilization, splicing [566], dendritic arborization [561], and it is also one of the primary stress granule proteins active in stress response [567]. In the healthy brain, tau participates in translational stress response by the promotion of stress granule (SG) formation, which allows adaptation of the protein synthesis and thus copes with stress. Under neuropathological conditions, TIA1 aggregates with pathological tau proteins and creates abnormal SGs; however, the clear consequences of these abnormal SGs are unknown [561]. Tau and TIA1 have a reciprocal effect on themselves. Tau can facilitate the assembly of TIA1 stress granules, and TIA1 facilitates the aggregation of phosphorylated and altered tau [568], and the binding of TIA1 to tau oligomers promotes their stabilization and accumulation. Furthermore, reduction of TIA1 levels prevents tau oligomers propagation and toxicity. TIA1 regulates the toxicity of tau oligomers by the determination of their amount and thus the response of neurons to toxic tau oligomers by binding them into stress granules [569]. However, TIA1 can also stabilize prefibrillar tau aggregates and inhibit their further assembly into large fibrils [570].

### 3.10. Signal Transduction Mediators Interacting with Tau

Signaling pathways in the cells regulate many different, important, and basic processes, like cell growth, proliferation [571], metabolism, cell-to-cell communication, regeneration [572], apoptosis, and stress response. Signaling pathways comprise four categories of components: surface or intracellular receptors, enzymes, transcription factors, and signal mediators [573,574]. In tauopathies, changes in signaling pathways occur, such as the cell cycle pathway, response to stress, and Ca^2+^ signaling pathway. For example, oxidative stress in neurons can provoke stress responses and induce several signaling pathways, like the stress-activated protein kinase pathways (JNK-SAPK and p38-SAPK2). These pathways result in either stress adaptation or apoptosis, thus their outcomes can be neuroprotective or neurodegenerative [575]. Changes in the Ca^2+^ signaling pathways occur in AD, where they lead to progressive decline in memory and apoptosis [576]. Currently, eleven proteins that are components of signaling pathways were experimentally confirmed as TIPs.

The 14-3-3 adaptor proteins regulate a wide range of pathological and physiological processes [577]. They are involved in neuronal migration, neuromorphogenesis, synaptogenesis, and development of the nervous system, including neurogenesis and differentiation [578]. They also participate in neurodevelopmental disorders by the regulation of the subcellular localization and activity of target proteins [579]. In pathological conditions, they can participate in the development of neurodegeneration or can be neuroprotective. For example, 14-3-3 zeta facilitates GSK3β-dependent tau phosphorylation by the affinity enhancement of GSK3β for tau [580]. 14-3-3 zeta-bound proteins are also resistant against protein phosphatases, which enhance the strength and duration of kinase-dependent signals under pathological conditions. The 14-3-3 proteins bound to tau favor tau phosphorylation and aggregation [581]. They were found also in NFT [582]. However, they also play a neuroprotective role by inhibiting the cell cycle activation and preventing neuronal death [583]. A more comprehensive description of the interaction of 14-3-3 proteins with tau was recently reviewed by Chen et al. [584].

The next TIP involved in signaling is the growth factor receptor-bound protein 2 (GRB2) [235], an adapter protein that plays a key role in Ras-mediated growth factor signaling, proliferation, and cell cycle. GRB2 also presents an important link between cellular signaling and neuronal cytoskeleton [235]. In a healthy brain, GRB2 is localized in the neuronal body and its projections, while in the AD, it disappears from projections and is restricted only to neuronal soma [585]. GRB2 interacts with tyrosine-phosphorylated APP in AD [586]. GRB2 is involved in the activation of the mitogen-activated protein kinase (MAPK) pathway leading to abnormal tau phosphorylation [587]. However, the direct consequence of tau interaction with GRB2 is unknown.

The prosaposin receptor Gpr37-like 1 (GPR37L1) [77] is the G-protein coupled receptor exclusively expressed in the nervous system and is abundant in the human brain [588]. It possesses functional similarity to the prosaposin receptor GPR37 (GPR37), which is a substrate for E3 ubiquitin-protein ligase parkin. Both receptors, GPR37 and GPR37L1 bind the neurotrophic protein prosaposin and its active fragment prosaptide, which causes the endocytosis of both receptors upon binding and activation of the ERK phosphorylation pathway. Prosaposin as the neurotrophic factor promotes cell survival, neurite outgrowth, and differentiation [589]. One study revealed that GPR37L1 is a constitutively active receptor-independent from prosaposin binding and that its activity is down-regulated by ADAM metalloprotease [590]. The GPR37L1 was only recently identified as TIP [77], however, the consequence of its interaction with tau protein has not been elucidated.

Hepatocyte growth factor-regulated tyrosine kinase substrate (HGS) [591] is involved in intracellular signal transduction mediated by cytokines and growth factors and is a part of the ESCRT-0 sorting complex. HGS is essential for the binding of ubiquitylated cargo into multi-vesicular bodies (MVBs), which are important for the lysosomal recycling of plasma membrane proteins [592]. Furthermore, HGS is also an effector of the small GTPase Rab35, which catalyzes the ESCRT-0 complex recruitment and MVBs formation [593]. The Rab35 and the ESCRT-0 machinery are active in the regulation of total tau turnover and specific phospho-tau species by lysosomal degradation since tau proteins are present in HGS-positive early endosomes [591]. In AD, impairment of tau proteostasis occurs, which is linked to neuronal and synaptic dysfunction. In the early stages of AD, abnormalities of the endolysosomal pathway, like high levels of lysosomal hydrolases and endosomal enlargement [594], and dysregulation of the hypothalamic-pituitary-adrenal axis occur, which lead to elevated glucocorticoid levels [595]. Moreover, the high glucocorticoid levels impair tau degradation by the downregulation of Rab35 expression, which suppresses the sorting of tau into the ESCRT pathway and the formation of the HGS-positive endosomes containing tau. In addition, HGS levels are significantly decreased in AD, which suggests that molecular components critical for membrane protein sorting in the endocytic pathway are altered [596]. This results in the accumulation of ubiquitinated tau and related neuronal death [591].

JNK-interacting protein 1 (JIP1) mediates the JNK signaling and MAPK kinase cascade activation. JIP1 binds to the kinesin-I motor complex and regulates the release of cargo from the kinesin at the transport destination. Phosphorylated tau interacts with JIP1 and re-localizes it from axons to neuronal soma. This interaction was shown to be pathological because it competes with the binding of JIP1 to kinesin leading to failure of JIP1-mediated phosphorylation of the kinesin motor complex and damage of the anterograde axonal transport [597].

LIM and senescent cell antigen-like-containing domain protein 1 (LIMS1) are required for the formation of multi-protein complexes and facilitate cell proliferation, migration, and survival [598]. During neuronal development, LIMS1 is necessary for the maintenance of neuronal polarity and communication in synapses [599]. In a healthy brain, the levels of LIMS1 are nearly undetectable, while during neurodegeneration it is robustly expressed [600]. In the study by Ozdemir et al., authors propose that LIMS1 plays a role in tau hyperphosphorylation and the stabilization of abnormally hyperphosphorylated tau [598].

The phospholipase C-gamma-1 (PLCG1) is essential for the production of diacylglycerol and inositol 1,4,5-triphosphate, the second messenger signaling molecules [601]. PLCG1 participates in the pathways of growth factor neutrophins in neurons and is involved in the development of the brain, cytoskeleton organization, and synaptic plasticity [602]. PLCG1, along with protein kinase D1, participate in the neuroprotective function of APOE-containing lipoproteins, through anti-apoptotic signaling [603]. In AD, PLCG1 levels are lower than in a healthy brain [604]. Tau protein interacts directly with the SH3 domain of PLCG1 by the PXXP motif in the proline-rich region and this interaction is regulated by phosphorylation of tau [235]. This suggests that tau may be involved in the regulation of signal transduction mediated by PLCG1.

## 4. Current Therapeutic Approaches Targeting Tau and Its Interacting Partners

The modulation of the interaction between Tau and its partners has been applied for the development of therapeutics for AD and PSP. The modulators in clinical trials can be divided into four groups, such as inhibitors of phosphorylation, acetylation, and de-glycosylation and molecule-activated dephosphorylation. The drugs in clinical studies and their outcome are summarized in Table 5.

Several more drugs which have TIPs as targets are currently in the preclinical phases of development. Most of them have the same targets as modulators in clinical trials. Among them are inhibitors targeting Cdk5 kinase [619], two inhibitors of caspases [620,621,622], several HDAC6 specific inhibitors [623,624,625,626], and modulators of chaperones Hsp90 [627] and Hsp70 [628,629]. All of them showed modulation of tau protein modifications (phosphorylation, acetylation, etc.) and thus influencing their interaction with tau protein.

## 5. Conclusions

The exact causes of tau pathology in Alzheimer’s disease (AD) and other tauopathies have not been well understood and are under the broad current research. Besides tau pathology, the next widely accepted model of AD progression is the Aβ hypothesis, which is considered to be upstream of tau pathology by many researchers [630,631]. However, other insights on the initiation of tau pathology have been proposed, such as the impaired cholesterol metabolism [632], deregulated endocytosis [473,633], or overactivated microglia [634]. On the other hand, the pathological accumulation of tau proteins well correlates with the cognitive decline in individuals suffering from AD [635]. Furthermore, the recently developed positron emission tomography (PET) tracers raised against Aβ and tau, showed that both types of pathologies initially start in different brain regions [636,637]. Moreover, it is suggested that the accumulation of the Aβ occurs 20–30 years before the clinical onset of AD [638]. These seemingly contradictory facts demonstrate the need for a better understanding of the precise molecular mechanisms of upstream pathological processes in AD and other tauopathies.

Since protein-protein interactions (PPIs) represent a core part of each molecular pathway and biological process [639], it is important to study the interactome of proteins involved in diseases such as AD. Key players of affected molecular pathways could be suitable targets for drug development and disease-modifying therapies. The pathological modifications of tau protein which include abnormal phosphorylation, truncation, acetylation, glycosylation, or ubiquitination are performed by various classes of enzymes interacting with tau. Therefore, it is proposed that interaction partners of tau protein and their corresponding molecular pathways may be responsible for the development of pathological forms of tau [640]. In this review, we summarized the current knowledge about the role of tau protein in physiology and pathology and reviewed the TIPs. We discussed the functions of individual TIPs in neurodegeneration and pointed out the several discrepancies and gaps in knowledge of their roles in tau pathology. We believe that this review will encourage basic research on TIPs and/or the targets for the development of effective therapies against tauopathies.

## Figures and Tables

**Table 1:** Overview of proteins and substances found mounted in paired helical filaments and neurofibrillary tangles.

Category	Proteins/Substances	Reference
Tubulin interacting proteins	microtubule-associated protein 2	[122]
	microtubule-associated protein 1B	[123,124]
	neurofilament proteins, vimentin	[125]
	heparansulfate proteoglycans	[126,127]
	amyloid precursor protein	[128,129]
Kinases and other cytosol enzymes	casein kinase II	[130]
	extracellular signal-related kinase-2	[131]
	glycogen synthase kinase-3	[132]
	phospholipase C-δ	[133]
Stress molecules	advanced glycation end products	[134,135,136]
	malondialdehyde	[137]
	heme oxygenase-1	[136]
Amyloid and amyloid-binding proteins	complement proteins (Clq, C3, C4d and C5b-9)	[138,139]
	vitronectin	[140]
	apolipoprotein-E	[141,142]
Others	C-series of gangliosides	[124]
	ubiquitin and ubiquitin ligases	[143,144,145]
	synaptophysin	[146]
	anti-thrombin III	[147]
	lactotransferrin	[148]

**Table 2:** Overview of protein kinases interacting with tau protein and their roles in pathology.

Protein	Roles in Tau Pathology	References
ABL1	involved in formation of apoptotic complex	[172]
AKT1	neuroprotective activity; inactivates pro-apoptotic proteins and promotes cell survival	[173]
BRSK (1, 2)	unknown role in tau pathology	-
CAMK2A	involved in hyperphosphorylation of tau in AD	[174,175]
CDK (1, 5)	accelerate tau hyperphosphorylation and formation of NFT; act neuroprotective by apoptosis inhibition	[176,177]
CDK2	inhibits tau-mediated tubulin polymerization by tau phosphorylation	[178]
CSNK1 (A1, D)	involved in formation of Aβ; colocalize with NFT, suggesting a role in tau aggregation	[179,180]
DYRK1A	overexpressed in AD; facilitates further tau phosphorylation by GSK3B	[181,182]
DYRK2	overexpressed in AD; involved in activation of apoptosis	[181,183]
FYN	facilitates formation of NFT by tau hyperphosphorylation; activates PTK2B; potentiates Aβ-induced synapse damage	[184,185,186]
GSK3 (A, B)	responsible for hyperphosphorylation of tau; involved in activation of apoptosis; exacerbate tau pathology when activated by Aβ	[187,188,189]
CHEK (1, 2)	unknown role in tau pathology	-
LCK	it is downregulated in AD; unknown role in tau pathology	[190,191]
LRRK2	contradictory reports: enhances abnormal tau phosphorylation and promote tauopathy or, no impact on tau pathology was shown	[192,193,194,195]
MAP2K7	colocalize with hyperphosphorylated tau; contribute to Aβ accumulation	[196,197]
MAPK (1, 3, 8–14)	overactivated in AD; involved in abnormal tau phosphorylation	[198,199]
MARK (1–3)	phosphorylate tau aggregates; colocalizes with NFT	[200]
MARK4	phosphorylate tau aggregates; mutation causes tau hyperphosphorylation, aggregation and tau-mediated neurodegeneration; colocalizes with NFT	[200,201]
PHKG1	unknown role in tau pathology	-
PKN1	regulates tau phosphorylation; colocalize with NFT	[202,203]
PRKACA	abnormally phosphorylate α-Synuclein-bound tau; facilitates further phosphorylation of tau by GSK3 kinase; colocalize with NFT; important for regulation of alternative splicing of exon 10 in MAPT	[204,205,206,207]
PRKC (A, B, E, G, I, Z)	neuroprotective role through inhibition of GSK3 kinase and reduced tau phosphorylation	[208,209]
PTK2B	hyperphosphorylates tau; is regulated by FYN kinase; colocalize with pathological tau proteins in brains of AD patients	[185,210]
RPS6K (A1, A3, A5, B1)	their attenuation is neuroprotective; possibly upregulate tau translation; contribute to tau aggregation and synapse damage; inhibit apoptosis	[211,212,213]
SGK1	upregulated in AD; enhances tau hyperphosphorylation by GSK3B activation	[214]
SIK1	unknown role in tau pathology	-
SRC	unknown role in tau pathology	-
SRPK (1, 2)	involved in regulation of alternative splicing of exon 10 in MAPT, where impaired splicing of exon 10 can lead to tauopathy	[215,216]
SYK	accumulation of pathological tau proteins cause overactivation of SYK, which further hyperphosphorylates tau and exacerbate pathology; contributes to the neuroinflammation; inhibits autophagic tau degradation; contributes to neuronal loss	[217,218,219]
TTBK1	enhances tau phosphorylation and aggregation; involved in neurodegeneration	[220,221]

**Table 3:** Overview of protein phosphatases interacting with tau protein and their roles in pathology.

Protein	Roles in Tau Pathology	References
PPP1C (A, B, C)	downregulated in AD; neuroprotective function by reducing hyperphosphorylated tau levels;	[170,222]
PPP2C (A, B)	downregulated in AD what induces tau hyperphosphorylation; neuroprotective; regulate autophagic degradation of proteins	[170,222,223,224]
PPP5C	downregulated in AD what contributes to increased levels of hyperphosphorylated tau; dephosphorylates AD phospho-tau; protects against Aβ toxicity	[225,226,227]
PTPN11	upregulated in AD; unknown role in tau pathology	[228]

**Table 4:** Overview of proteins participating in the regulation of kinases or phosphatases known to interact with tau protein and their roles in pathology.

Protein	Roles in Tau Pathology	References
PTPA	regulatory subunit of PPP2C (A, B); decreased expression in AD; enhances tau dephosphorylation; neuroprotective; regulate autophagic degradation of proteins	[223,224,229,230]
CDC37	regulates tau phosphorylation, enhances phospho-tau stability at HSP90 scaffold, preventing its degradation, preserves CDK5 and AKT kinases	[231]
PIK3R1	key unit of PI3K, part of the PI3K/AKT1/GSK3B signaling pathway, which is downregulated in AD, plays a role in insulin signaling pathway where it might exacerbate AD pathology	[232,233,234,235]
S100B	inhibits tau phosphorylation; enhances tau dephosphorylation by PPP5C; overexpression of S100B causes apoptosis, tau hyperphosphorylation, and inflammation	[236,237,238,239,240]

**Table 5:** Compounds in clinical trials targeting tau interactions with its PPIs.

Name	Function	Phase (Disease)	Efficacy	Source
Dasatinib	Ab1 and Src kinase inhibitor	Phase I and II	Recruiting	NCT04063124
Epigallocatechin gallate	DYRK1A kinase inhibitor	Phase II (AD)	No results posted	NCT00951834
Lithium chloride	GSK-3 kinase inhibitor	Phase II (AD/PSP)	No effect	NCT00088387 [605]
Nilotinib	Ab1 kinase inhibitor	Phase II (AD/PD)	Unknown	NCT02947893 [606]
Saracatinib	Ab1, Src kinase inhibitor	Phase II (AD)	No effect	NCT02167256 [607,608]
Tideglusib	GSK-3 kinase inhibitor	Phase II (AD/PSP)	No effect	NCT01350362 [609,610,611,612]
Valproate	GSK-3 kinase inhibitor	Phase III (AD)	No effect	NCT00071721 [613,614,615]
Memantine	PPP2CA activator	Phase IV (AD)	No effect	NCT00469456 [616]
Sodium selenate	PPP2CA activator	Phase II (AD)	Small positive effects	[617]
Salsalate	Acetylation inhibitor	Phase I (AD/PSP)	Ongoing	NCT03277573 [618]
Minocycline	CDK5 kinase/caspase-3 inhibitor	Phase II (AD)	No effect	NCT01463384
Nicotinamide	HDAC inhibitor	Phase II	Recruiting	NCT03061474
Phenylbutyrate	HDAC inhibitor	Phase II	Ongoing	NCT03533257
Vorinostat	HDAC inhibitor	Phase I	Recruiting	NCT03056495

## Data Availability

Not applicable.
